# Intersectoral and multisectoral approaches to enable recovery for people with severe mental illness in low- and middle-income countries: A scoping review

**DOI:** 10.1017/gmh.2023.10

**Published:** 2023-03-15

**Authors:** André J. van Rensburg, Carrie Brooke-Sumner

**Affiliations:** 1 Centre for Rural Health, University of KwaZulu-Natal, Durban, South Africa; 2 Alcohol, Tobacco and Other Drug Research Unit, South African Medical Research Council, Cape Town, South Africa

**Keywords:** recovery, severe mental illness, community-based initiatives, collaborative care, developing countries

## Abstract

The needs of people with severe mental illness are complex and require a range of services embedded in well-coordinated systems of care to enable recovery, promote well-being and optimise social integration. The concept of recovery is strongly rooted in the centrality of multi and intersectoral systems of care, and, while multi and -intersectoral dimensions of mental health systems have been highlighted in analyses focusing on high-income regions, little has been elaborated in terms of these approaches in the recovery of people with severe mental illness (SMI) in low- and middle-income countries (LMICs). The aim of this review was to identify and describe multi and intersectoral approaches underpinning community-based SMI recovery interventions in LMICs. A scoping review was carried out following the following steps: (1) Objectives for the review were developed and refined; (2) A systematic search of databases (EbscoHost, PubMed, Google Scholar) and previous reviews were undertaken from 2012 to 2022, where relevant papers were identified; (3) Papers with a focus on SMI and recovery, a specific description of an intervention, located in LMICs, with explicit linkages between sectors, and published in English, were selected for inclusion; (4) Data were extracted and charted and (5) Findings were analysed and reported thematically. Thirty-six papers were included for analysis, from 18 countries, including qualitative studies, trials, desktop and secondary data reviews and case studies. Examples of multi- and intersectoral action included collaboration between healthcare and community support systems, collaboration in providing supported housing and supportive community spaces for recovery, and linkages between biomedical and social spheres of care. Barriers included the dominance of mental health professions in delivering care, community-based stigmatising attitudes towards SMI. Multi- and intersectoral collaboration for SMI recovery requires investments in financing, education and coordination by a governing body.

## Graphical abstract

**Figure:**
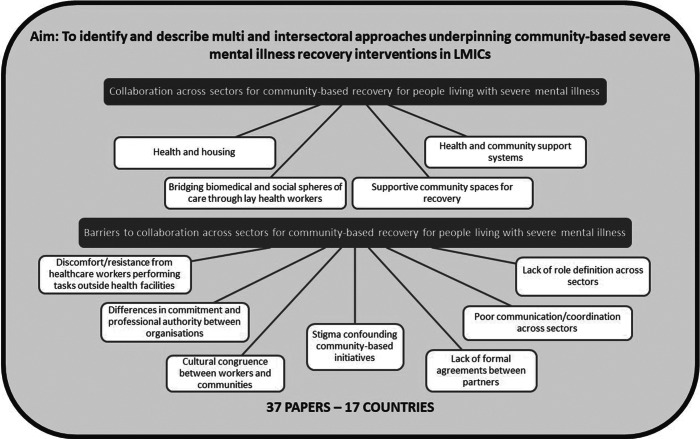

## Impact statement

Despite a large body of work on recovery for people living with severe mental illness (SMI), and its implicit embeddedness in collaboration across sectors, little systematic description has been undertaken of its implementation in low- and middle-income countries. Our review fills this gap by providing a synopsis of how multi- and intersectoral collaboration in supporting recovery occur in these contexts. It highlights examples that involve collaboration between healthcare and community support systems, collaboration in providing supported housing and supportive community spaces for recovery, and linkages between biomedical and social spheres of care. There are, however, barriers to collaborating across sectors, including the dominance of mental health professions in delivering care, community-based stigmatising attitudes towards SMI, and a discomfort of some healthcare workers to work beyond the professional boundaries of healthcare. Multi- and intersectoral collaboration for SMI recovery needs to be driven by formal structures and financing, including both on macro and micro levels of engagement.

## Introduction

People living with severe mental illness (SMI) have substantially increased relative mortality risk compared to the general population, related to cardiovascular disease (Ali et al., 2022; Lambert et al., 2022), and in low- and middle-income countries (LMICs) particularly related to poverty that leads to poor health status (e.g., undernutrition) (Jenkins et al., 2011c; Tirfessa et al., 2019). While symptoms of the illness play a role in course and outcomes, globally and in LMIC particularly, people with SMI may experience social and economic adversities and human rights abuses that can create a social environment that hampers clinical and personal recovery (Brooke-Sumner et al., 2014; Patel, 2016; Asher et al., 2017). Recovery, as it has been conceptualised in high-income country (HIC) settings, is described as an individual journey of transformation and personal growth moving from the distress of the acute experience of the condition towards finding meaning and purpose, a sense of belonging, forming or rebuilding meaningful relationships (Frost et al., 2017), bringing hope, empowerment, goal orientation and fulfilment (Warner, 2009; Drake and Whitley, 2014; Whitley et al., 2015). Recovery encompasses concepts of prosperity (legal, political and economic dimensions); individual recovery (dimensions of normalcy, knowledge, individuality, responsibility and identity); clinical recovery (treatment and diagnosis dimensions) and social recovery (externally and internally derived notions of social awareness, being a part of society, functioning well within, groups, treated as an equal) (Vera San Juan et al., 2021). Biomedically oriented health systems alone are inadequately configured to address the spectrum of these recovery needs which extend across intersecting social, economic, cultural and political spheres, beyond the health sector (Gamieldien et al., 2022). While many of the recovery concepts may be cross-cutting among HIC and LMIC, some concepts, developed in Western sociocultural contexts, may be limited in being rooted in economic environments and health and social welfare systems able to provide for people’s material needs (Gamieldien et al., 2021). In LMICs there may be greater involvement of families in providing care and supportive environment, use of non-Western healing approaches (Onken et al., 2007), and a more important role of spirituality in recovery (Gamieldien et al., 2021).

Since its introduction into health policy discourse in the 1970s, “intersectoral action” has become a staple in framing responses to public health challenges. The need for the health sector to collaborate with a range of other sectors to improve health outcomes continues to be highlighted (Sanni et al., 2019). More recent conceptualisations include “multisectoral action for health” which refers to the deliberate or collateral inclusion of different actors and sectors in health improvement, including initiatives such as “Whole of Government,” “Joined-up Government” approaches, horizontal and integrated policymaking, and Health in All Policies. Despite the conceptual promise of inter-and multisectorality, and evidence of its implementation in HIC (Diminic et al., 2015; Jørgensen et al., 2020; Jørgensen et al., 2021; Mondal et al., 2021) this has not consistently translated into policy or services. For instance, neither the WHO’s Innovative Care for Chronic Conditions Framework (Nuño et al., 2012), nor its subsequent modification for LMICs or countries in health transition (Oni et al., 2014), adequately considers the role of sectors outside of health. A well-documented example of the costs of failure to approach community mental health from an intersectoral approach is the US deinstitutionalisation movement. Following the policy shifts towards deinstitutionalisation, financial costs and responsibilities were dispersed through various stakeholders and agencies. This led to a fractured system, inadequate to address the complex needs of people with SMI, leading to homelessness or incarceration when placed in community settings (Grazier et al., 2005). In order to develop more people-centred, humane and effective community mental health systems, recovery should be firmly couched in service and strategic collaboration across sectors (Drake and Whitley, 2014). Several examples of promising shifts towards intersectoral collaboration in community SMI services have emerged in high-income settings. Intersectoral service networks in Belgium (Nicaise et al., 2021) and Canada (Fleury et al., 2017) includes integrated, intersectoral collaboration in the form of housing, educational and employment support, beyond medical and psychiatric care. The Australian Partners in Recovery model is a good example of how care coordination can aid recovery for people living with SMI (Isaacs, 2022). A review of interventions that focus on system-level intersectoral linkages involving mental health services and non-clinical support services yielded 40 examples from HICs, with various different collaboration modalities. Outcomes reported were largely positive, particularly regarding improved interagency communication, mutual understanding and empathy, cost efficiency, involvement of lay health workers, as well as various service user outcomes such as clinical functioning, employment prospects and accommodation stability (Whiteford et al., 2014). This being noted, the connection between recovery and intersectoral care remains relatively ill-defined and several gaps remain in this body of evidence (Jørgensen et al., 2021). However the relevance and need for development of this approach to recovery services are highlighted in the 2022 World Mental Health report (World Health Organization, 2022).

While there is much promise of inter- and multisectoral approaches to SMI recovery, there is paucity of reviews on the subject – particularly in LMICs, and a lack of systematised evidence on how to implement the approach. While the implementation of intersectoral collaborations to enable recovery of people living with SMIs have been well-described in HICs, it remains uncertain how intersectoral care is being pursued in contexts faced with a lack of resources and infrastructure, mental health system investment-to-population ratio, substantial geographical and cultural variation and underdeveloped welfare systems (Patel, 2016). The aim of this scoping review was therefore to identify and describe multi and intersectoral approaches underpinning community-based SMI recovery interventions in LMICs.

## Methods

This scoping review was guided by the methodological steps outlined by Arksey and O’Malley (2005) and the Johanna Briggs Institute (JBI, 2015), following the following phases: (1) Objectives for the review were developed and refined among the authors, based on a brief, initial literature review; (2) A systematic search of databases was undertaken where relevant papers were identified; (3) Relevant papers were selected for inclusion; (4) Data were extracted from these selected studies, and were charted according to the Preferred Reporting Items for Systematic reviews and Meta-Analyses Extension for Scoping Reviews (PRISMA-ScR) (Tricco et al., 2018) and (5) Findings were thematically analysed and reported.

The search was undertaken by the authors, with weekly discussions to compare results and discuss inclusions and exclusions. We applied search terms as used in a recent scoping review exploring recovery of people living with SMI in LMICs (Gamieldien et al., 2021), with updated time parameters to reflect our search scope of 2012–2022. This resulted in an additional 12 papers added to their results (22 in total). We then conducted searches using key terms related to recovery, SMI, community settings, LMICs (see Supplementary Material for a full description of search terms), in EbscoHost (Academic Search Complete; APA PsycInfo; Health Source – Consumer Edition; Health Source: Nursing/Academic Edition; MasterFILE Premier; MEDLINE with Full Text), PubMed and Google Scholar. In Google Scholar, the terms and related terms “recovery,” “SMI,” “community settings,” and “LMICs” were included and results were screened until two sequential pages did not yield any further papers that adheres to the inclusion criteria. During the screening and review process, it became apparent that the interchangeable and ambiguous application of complex terms such as recovery, multi-and intersectoral approaches, may limit the number of papers found in databases. Therefore, an additional review of the results of 11 systematic reviews on psychosocial interventions with a focus on SMI was undertaken (Brooke-Sumner et al., 2015; Lutgens et al., 2017; Sin and Spain, 2017; Davies et al., 2018; Frederick and VanderWeele, 2019; Alhadidi et al., 2020; Al-Sawafi et al., 2020; Bighelli et al., 2021; Morillo et al., 2022; Rodolico et al., 2022; Solmi et al., 2022), while peer reviewers helpfully pointed out additional omissions in the results. This underlines the importance of including an additional consultation phase in scoping reviews, framed as an optional step in existing guidelines (Levac et al., 2010). Inclusion and exclusion criteria are summarised in Table 1.

**Table 1.** Inclusion and exclusion criteria

Inclusion criteria	Exclusion criteria
A primary focus on people living with SMI, as defined by the National Institute for Mental Health (“severe” and “serious” were used interchangeably), that is, “…a mental, behavioural, or emotional disorder resulting in serious functional impairment, which substantially interferes with or limits one or more major life activities. The burden of mental illnesses is particularly concentrated among those who experience disability due to SMI.” (National Institute of Mental Health, 2022). This includes specific ICD-10 diagnostic categories F20–29, F30–39, F30.2, F31.2, F31.5, F32.3, F33.3	Primary focus not on people living with SMI
A description of a specific intervention, programme or service	General overviews or descriptions of health systems or services, rather than a specific focus on an intervention aimed at recovery from SMI
A focus on community-based, outpatient settings	A focus on inpatient, institutionalised settings
A focus on the enabling of recovery as defined in the Introduction	No focus on dimensions of recovery
Adults (aged 18 years and above)	People under 18 years of age
Studies reported during the past decade (2012–2022)	Studies reported before 2012
Studies with a primary location in LMICs (World Bank, 2022)	Studies focusing on settings in HICs (World Bank, 2022)
Explicit description of intended linkages with sectors other than health	No apparent linkages of an intervention with sectors beyond health
Published in English	Full text not in English

## Findings

### Search results

**Figure 1.**
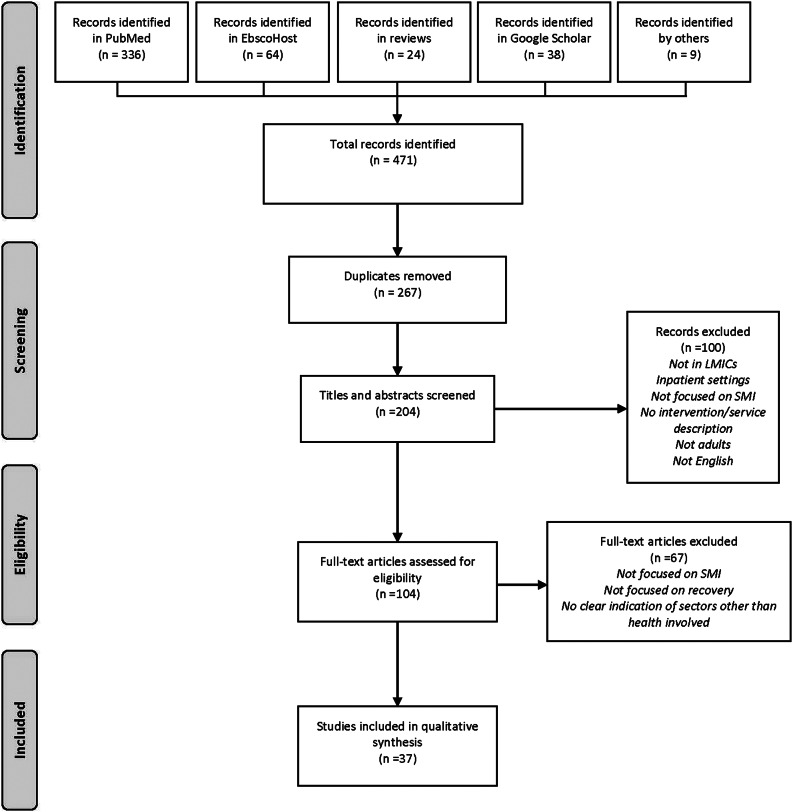
PRISMA illustration of search and selection process.

As shown in Figure 1, a total of 471 papers were initially identified through PubMed (*n* = 336), EbscoHost (*n* = 64), Google Scholar (*n* = 38), results of other reviews (*n* = 24) and peer reviewers (*n* = 9). After duplicates were removed, 204 titles and abstracts were screened, where 100 records were excluded based on the exclusion criteria. Two papers were excluded due to language, one was in Turkish, the other in Portuguese. Following a screening of 104 full-text papers, an additional 67 papers were excluded due to not having a primary focus on SMI, no clear focus on recovery, and no apparent linkages with sectors other than health. This resulted in 37 papers being included for qualitative synthesis (Table 2).

**Table 2.** Overview of included studies

Citation	Country	Primary intervention	Dimensions of involvement of different sectors in recovery	Study type	Data type
Acebal et al. (2021)	Brazil	Psychosocial rehabilitation	Community-based Residential Therapeutic Services (SRT) in conjunction with routine medical care	Cross-sectional survey	Quantitative
Anish (2013)	India	Psychosocial rehabilitation	Collaboration between healthcare and agriculture industries to promote recovery through vocational activities in diary and farming, and horticulture	Programme evaluation	Quantitative
Arahanthabailu et al. (2022)	India	Assertive community treatment	Manipal Assertive Community Treatment (M-ACT) teams liaise with community resources to facilitate vocational rehabilitation and access to welfare benefits	Quasi-experimental	Quantitative
Arias et al. (2016)	Ghana	Prayer camps	Collaboration between biomedical services and faith-based care delivered at prayer camps	Exploratory qualitative study	Qualitative
Asher et al. (2015), Asher et al. (2022)	Ethiopia	Community-Based Rehabilitation Intervention for People with Schizophrenia	Using community-based rehabilitation workers as principal deliverers of the intervention allows networking and integration with NGOs and traditional health practitioners	RCT	Quantitative and qualitative
Brooke-Sumner et al. (2016)	South Africa	Intersectoral psychosocial rehabilitation	Despite very little formal collaboration between government departments, singular examples emerged, for example, collaboration between social development and public works in placing people living with schizophrenia in an employment programme	Exploratory qualitative study	Qualitative
Brooke-Sumner et al. (2018)	South Africa	Psychosocial rehabilitation (group-based, psychoeducation)	Psychosocial rehabilitation programme delivered by auxiliary social workers via collaboration between the health and social development/welfare sectors	Quasi-experimental	Mixed methods
Chatterjee et al. (2014)	India	Collaborative community-based care	Collaborative package of community-based care facilitated linkages between service users and (1) user-led support structures, (2) community support agencies to address social issues and improve social inclusion and (3) community agencies that provide legal and employment services	RCT	Quantitative
Chen et al. (2020)	China	Psychosocial rehabilitation (clubhouse model)	The Clubhouse facilitates transitional employment and supported education programmes in collaboration with community partners	RCT	Quantitative
de Menil et al. (2015)	Kenya	Mental Health and Development model	Collaboration between the Ministry of Health, NGOs, the Ministry of Gender, Children and Social Services, and Ministry of Agriculture, Livestock and Fisheries, to provide a continuum of services including self-help groups and training in and support for livelihood and farming capacities	Cost-effectiveness analysis	Primary and secondary quantitative data
Gamieldien et al. (2022)	South Africa	Perceptions on recovery	NGOs provide multisectoral services to aid recovery efforts, including basic needs, transportation, life skills, vocational training and leisure and sport activities	Exploratory qualitative study	Qualitative
Hall et al. (2019)	Timor-Leste	Intersectoral mental health service collaboration	Referral linkages between government health facilities, police, local authorities, private clinics, social sector service providers, and customary healers, forming a network of services that include health care, disability support, victimisation support and residential support	Case study	Mixed methods
İncedere and Yildiz (2019)	Turkey	Case Management for Individuals with Severe Mental Illness	A case manager liaises with employment sector to identify suitable candidates to undertake an examination and be positioned for an appropriate job	Quasi-experimental	Quantitative
Janse Van Rensburg et al. (2018)	South Africa	Service referrals between state and non-state actors	Government clinics and hospitals refer service users to NGOs for residential support and welfare grant application assistance	Case study	Mixed methods
Kallivayalil and Sudhakar (2018)	India	Low-cost community psychosocial rehabilitation model	NGO has a candle-making unit where people living with severe mental illness can sell candles at nearby churches during holy days, while clothes manufacturing initiative linked service users up with retail outlets to sell clothes	Quasi-experimental	Survey
Kohrt et al. (2015)	Liberia	Crisis Intervention Team (CIT) Model of Police–Mental Health Collaboration	The Carter Center Mental Health Program (TCC-MHP) facilitated partnerships to advance mental health policy, legislation, and funding, which included engaging with the Liberia National Police to identify spaces for collaboration on crisis intervention	Programme development description	Narrative description
Li and Ma (2021)	China	National comprehensive management pilot project for integrated care for people with severe mental disorders through strengthened cooperation among government organisations and between government and other relevant social organisations	A quasi-governmental organisation establishes and coordinates community service organisations for people living with severe mental illness, with formal links between the ministries of health and social affairs. Through training and cooperation across a range of organisations and sectors, an integrated package of services is provided to be more responsive to individual needs	Case study	Qualitative interviews
MacDougall et al. (2022)	Kenya	Community REcovery Achieved Through Entrepreneurship (CREATE)	An initiative that integrates elements of psychosocial rehabilitation (PSR), community-based rehabilitation (CBR), and work integration social enterprise (WISE)	Programme development description	Qualitative
Mascayano et al. (2019), (2022)	Brazil and Chile	Critical Time Intervention with Task-sharing (CTI-TS)	Teams made up of auxiliary and peer workers supported service users following discharge from acute psychiatric hospitalisation, to facilitate linkages with a range of community-based support systems that included basic and specialist medical care, psychosocial rehabilitation, leisure and art-based activities and basic needs	RCT	Quantitative
Molchanova (2014)	Kyrgyz Republic	Indigenous model of family rehabilitation	The development of family-driven NGOs led to the provisioning of a range of psychosocial rehabilitation activities to people living with severe mental illness in community settings	Desktop review	Government documents
Muhić et al. (2022)	Bosnia and Herzegovina	Brief, multifamily group intervention for patients with schizophrenia and related disorders	Multifamily groups mobilised mutual support for people living with severe mental illness in community settings	RCT	Survey and qualitative interviews
Nxumalo Ngubane et al. (2019)	Eswatini	Psychiatric outpatient care	People living with severe mental illness’ engagement in community-based projects such as community kitchens for orphaned and vulnerable children aided in recovery efforts	Interpretive phenomenological analysis	Qualitative interviews
Padmakar et al. (2020)	India	The Banyan’s supported housing model	The Banyan organisation developed a supported housing programme where people living with severe mental illness can live independently, with an emergency care and recovery unit located in close proximity	Mixed methods	Qualitative interviews, logbook notes, survey
Pfizer and Kavitha (2018)	India	Interdisciplinary recovery model of psychosocial rehabilitation	People living with severe mental illness were enrolled into a sheltered workshop, where their functionality, occupational skills and readiness were improved and evaluated, after which appropriate service users could be promoted to peer mentorship at a trial worksite, with the ultimate goal of securing competitive employment. The programme also included generating financial support for the building of houses on private owners’ properties, as well as linkages with Alcoholics Anonymous to address substance abuse challenges	Desktop review	Narrative description
Raja et al. (2012)	Nepal	BasicNeeds model of Mental Health and Development	Livelihood support was provided to people living with severe mental illness and their families through cash grants or supporting the setting up of businesses, as well as support for the setting up of self-help groups	Case study	Project data
Rao et al. (2022)	India	SCARF Telepsychiatry in Pudukkottai (STEP) program	A range of community-based psychosocial rehabilitation activities was provided in a rural area, including facilitating access to disability and welfare benefits, supporting job-seeking efforts and facilitating placement in partner businesses, and supporting the obtaining of loans from banks to help set up small businesses	Desktop review	Project data
Rashed (2015)	Egypt	Qur’anic healing	A duality of recovery care that consisted of psychiatric services delivered by medical doctors, and Qur’anic healing providing spiritual care	Ethnography	Participant observation
Saha et al. (2020)	India	Non-governmental psychosocial rehabilitation centres	NGO that provided support for livelihood activities, access to government grant schemes, as well as a range of psychosocial therapies with service users and their families	Secondary data analysis	Patient case records
Soygür et al. (2017)	Turkey	Therapeutic community and supported-employment setting where people living with schizophrenia work	Blue Horse Café provides a protective space where people living with schizophrenia can work, build skills and develop independence from medical institutionalisation, while still accessing medical care	Phenomenological	Qualitative interviews
Subandi (2015)	Indonesia	Psychiatric outpatient care	In addition to outpatient community-based medical care, people accessed “natural therapy” in the form of spiritual guidance from mosques alongside neighbours and friends, which also allowed for community integration, while others accessed services from both biomedical and traditional health practitioners	Ethnography	Participant observations
Vijayan (2021)	India	Recovery Oriented Services (ROSeS)	Community-based psychiatric rehabilitation programme aids in recovery efforts by acting as an intermediary between service users and organisations that facilitate work placements	Case study	Qualitative
World Health Organization (2021)	Brazil	Centro de Atenção Psicosocial (CAPS)	Drives recovery efforts by facilitating active citizenship, which includes helping service users to navigate government bureaucracies to obtain formal documentation and access benefits, and liaising with a range of community resources to support housing, employment and social life improvement	Case study	Narrative description
World Health Organization (2021)	China	Phoenix Clubhouse	Clubhouse collaborates with business partners to facilitate placement for paid employment in the local labour market for its members	Case study	Narrative description
World Health Organization (2021)	India	Naya Daur Community Outreach	A community outreach programme that refers people to temporary shelters, where they can access basic services such as hygiene materials, food, water and a place to sleep	Case study	Narrative description
World Health Organization (2021)	India	Atmiyatab primary care community outreach service	Mental health champions are instituted as intermediaries for people living with severe mental illness and their families to access disability certification, to access government benefits such as pensions, grants and disability benefits, as well as work schemes	Case study	Narrative description
World Health Organization (2021)	Georgia	Hand in Hand supported living	An organisation providing employment support by collaborating with community social enterprises and employers	Case study	Narrative description
World Health Organization (2021)	India	Home Again housing and supportive services	The Banyan organisation’s housing and supportive services initiative provides access to housing, establishing work placements, educational support, and linking with various community resources to promote recovery efforts	Case study	Narrative description

### Included studies

An overview of included studies is presented in Table 2. Studies from an array of countries were included: Bosnia and Herzegovina (*n* = 1); Egypt (*n* = 1); Eswatini (*n* = 1); Ethiopia (*n* = 1); Ghana (*n* = 1); Indonesia (*n* = 1); Kenya (*n* = 1); Kyrgyz Republic (*n* = 1); Liberia (*n* = 1); Nepal (*n* = 1); Chile (*n* = 1); Timor-Leste (*n* = 1); Turkey (*n* = 2); Brazil (*n* = 3); China (*n* = 4); South Africa (*n* = 4) and India (*n* = 12). A variety of study designs and methodologies were reported, including various qualitative studies, randomised control trials, desktop and secondary data reviews, quasi-experimental studies and case studies.

### Overview of SMI recovery approaches

Several different approaches to supporting recovery were highlighted. A common initiative was the establishment of community-based psychosocial rehabilitation centres, which were often run as a collaborative between family members, mental health professionals, and other community resources, to provide psychosocial, job and basic needs support to people with SMI, described in India, Turkey and the Kyrgyz Republic (Molchanova, 2014; Soygür et al., 2017; Kallivayalil and Sudhakar, 2018; Pfizer and Kavitha, 2018; Saha et al., 2020). Also, the clubhouse model for psychosocial rehabilitation was reported in China (Chen et al., 2020), one case describing linkages with a supported employment programme in surrounding communities (World Health Organization, 2021). Supported housing, especially focusing on those experiencing poverty and homelessness, was described in India, (Anish, 2013; Padmakar et al., 2020; World Health Organization, 2021) and Brazil (Acebal et al., 2021). In some instances, mental health teams performed various services, for instance facilitating residential training and placement according to individual preferences and needs – an example is a Recovery Oriented Services (ROSeS) team in India facilitating placement at a rural development centre for an individual who was interested in agriculture and animal husbandry (Vijayan, 2021). Some teams, for instance, a mental health outreach team in India, also facilitated service access through telepsychiatry (Rao et al., 2022), and others, like Atmiyata, facilitated access to government-based social benefits including pensions, rural employment grants, disability benefits and other financial assistance, through the establishment of mental health champions (World Health Organization, 2021). Recovery models that included task-sharing of services to non-specialist workers were reported in South Africa (Brooke-Sumner et al., 2018) and India (Chatterjee et al., 2014). A Critical Time Intervention with task-sharing (CTI-TS) was reported in Chile and Brazil, involving psychosocial support during the transition from psychiatric hospital discharge to community settings (Mascayano et al., 2022). A case describing a multifamily group intervention based on trialogue, psychosis seminars, and co-learning was described in Bosnia and Herzegovina (Muhić et al., 2022), with an NGO-delivered multicomponent intervention for people with SMI and caregivers that included biomedical treatment and supporting economic independence in Nepal (Raja et al., 2012). The salience of integration with religious practices was described in Java (Subandi, 2015) and Egypt (Rashed, 2015). In China, a national pilot programme was described that involved psychosocial rehabilitation through cooperation among government organisations and between government and other relevant social organisations (Li and Ma, 2021). A study in Ethiopia seeking to develop a community-based rehabilitation intervention for people with schizophrenia, focused on the development of a specific cadre of worker that would facilitate better networking with other NGO services and expand to other forms of disability as well (Asher et al., 2015; 2022).

### Dimensions of multi- and intersectoral collaboration in supporting recovery

As suggested by the number of papers included in this synthesis, very few examples could be found that explicitly highlight the involvement of sectors other than health in recovery processes in community settings. Only one study described an intersectoral collaboration between health and other sectors in supporting SMI recovery on a national, policy-level scale, describing the formalising of governance and funding structures for better interorganisational collaboration and funding in China (Li and Ma, 2021). In terms of programmatic interventions, several dimensions of multi- and intersectoral collaboration emerged, described below in terms of Health and Housing, Health and Community Support Systems, Supportive Community Spaces for Recovery, and Bridging Biomedical and Social Spheres of Care through Lay Health Workers.

#### Health and housing

There were instances of collaboration between the health sector and various actors involved in providing supported housing to people living with SMIs. The Phoenix Clubhouse in Hong Kong, China, put in place arrangements with housing partners, including public housing, supported hostels, halfway houses, long-stay care homes and residential respite services, which members can access (World Health Organization, 2021). An Indian study (Anish, 2013) reported that the majority of residential facilities for people with SMI were provided by faith-based organisations with funds from public donations. These faith-based organisations tended to collaborate with other sectors during the period when service users are admitted to the centre following referral by mental health professionals, family members, police and social services.

An example of this kind of collaboration is the Banyan’s supported housing model. The Banyan started out as a crisis intervention and rehabilitation centre for homeless women with mental illness in the city of Chennai, India, and has expanded its services to include emergency, open shelter and street-based services, social care and long-term and alternative living. In support of living arrangements, the organisation entered into rental agreements with private property owners in order to secure housing for people with SMI, who were supported through stages of confrontation, adaptation and stabilisation (Padmakar et al., 2020). A Brazilian study (Acebal et al., 2021) investigated service users’ perspectives on the relationship between housing needs and mental health/illness. It highlighted the importance of the links between “residential therapeutic services” (supported housing) and biomedical health facilities but details of the working relationships between health facilities and residential facilities were lacking.

#### Health and community support systems

A key area for multi- and intersectoral collaboration is the setting up and strengthening of community-based support resources beyond the health sector. In the aforementioned CTI-TS model in South America, lay community mental health workers and peer support workers formed CTI teams that provided structured, time-limited support to people discharged from psychiatric hospitalisation. Working from community mental health centres, a key task in this initiative was to support beneficiaries through linking them to informal and formal support systems in communities (including local leisure clubs and community centres) after which a gradual withdrawal period would take place thereby lessening dependence on the CTI programme or institutional mental health services (Silva et al., 2017; Mascayano et al., 2019; Mascayano et al., 2022). The multifamily support group model in Bosnia and Herzegovina served to mobilise mutual support in community settings (Muhić et al., 2022), Two studies described mental health service networks across sectors, that included support for people living with SMI. In Liberia, The Carter Center Mental Health Program (TCC-MHP) partnered with the Liberian police sector to develop Crisis Intervention Teams (CIT) to create more supportive services for people living with SMI (Kohrt et al., 2015). In Timor-Leste, collaboration and referral between mental health and social service delivery platforms were reported, that included referral from police, local authorities, private care and social services to government health facilities for care, particularly those living with SMI. Government services in turn referred people to support organisations, including housing support for people living with SMI (Hall et al., 2019). In a similar study from South Africa, a range of NGO activities were described, where people living with SMI were sometimes referred to organisations for housing and basic needs support, as well as to a social services organisation that provided home-based psychotherapy, group therapy, social support, community awareness and education campaigns. There were also instances of collaborating with old-age facilities to provide housing support to people with SMI (Janse Van Rensburg et al., 2018).

#### Supportive community spaces for recovery

Given the history and prevalence of stigma, discrimination and structural barriers to social integration faced by people living with SMI, recovery processes require safe and supportive spaces in communities. The Centro de Atenção Psicosocial (CAPS) in Brazil is a network of community-based mental health centres, which promotes active citizenship through a range of services, including supporting people through the various bureaucracies of obtaining formal documentation and access social support, training and education, access to supportive housing and supportive work placement, with collaborations across the sectors of health, education, justice, social assistance and various non-governmental agencies (World Health Organization, 2021). The well-known clubhouse model of psychosocial rehabilitation, with its roots in the 1940s in New York, was applied in Chinese settings and involved non-residential services that included employment and supported education programmes, linked with private and education sectors (Chen et al., 2020; World Health Organization, 2021). Another example is the Blue Horse Café in Turkey, a therapeutic community and supported-employment setting where most services offered by the café are performed by people living with SMI, including food preparation and serving, reservation management, cleaning, management and organisation and selling of second-hand goods. This provides a protective environment within which people with SMI can participate in the labour sector, while also receiving therapeutic support (Soygür et al., 2017). Another programme in Turkey assisted service users through case management, where people were supported in preparing CVs and job interviewing, interviews with labour agencies, and reviewing of vacancies. People were also accompanied during job interviews, and during their first days of employment, and relationships were established between case managers and line managers in workplaces (İncedere and Yildiz, 2019). In India, the Rajah Rehabilitation Centre (RRC) collaborates with employers to secure employment for people with SMI, following a period of supported work and skills development. There is also collaboration with community-based Alcoholics Anonymous and Al-Anon support groups to support participants and their families who have to deal with challenges related to substance abuse, while legal services are made accessible through a collaboration with a Legal Aid clinic (Pfizer and Kavitha, 2018). The development of peer support networks in Kenya connected people living with SMI with skill-building in livelihood activities, such as drought-resistant farming and making detergent (de Menil et al., 2015). In Ghana, supportive spaces including housing were provided in prayer camps, overseen by local prophets (Arias et al., 2016).

#### Bridging biomedical and social spheres of care through lay health workers

Instances emerged where lay health workers were trained and supervised by mental health professionals to provide community-based services, thereby bridging the domain of healthcare within facilities with the social dimensions of recovery in community settings. In the community-based intervention for people with schizophrenia and their caregivers in India (COPSI), the programme included the linkage of people with SMI with community agencies and user-led self-help groups. This provided a support for seeking employment as well as to access social and legal benefits (Chatterjee et al., 2014). A similar intervention was described in South Africa, where a community-based psychosocial rehabilitation intervention was delivered in partnership with PHC health clinics and a local NGO by auxiliary social workers. Participants for the intervention were recruited through clinics and intervention conducted in clinic premises by auxiliary social workers (Brooke-Sumner et al., 2018). A Nepalese study of an NGO delivered multicomponent intervention for people with SMI and caregivers (access to biomedical treatment and enabling service users and caregivers to develop a livelihood) recommended expanding scope of training of community health workers to include skills in delivering support for sustainable livelihood interventions (Raja et al., 2012).

### Barriers to multi- and intersectoral collaboration

Though difficult to assess comprehensively due to the ambiguity of descriptions of multi- and intersectoral collaboration, limited barriers to such collaboration emerged. A key barrier highlighted in the Chinese interorganisational collaboration case was differences in commitment and professional authority between organisations, both government and non-government. Specifically, there stronger institutional commitment of actors in the health sector was reinforced by the greater degree of professional authority wielded by psychiatrists (Li and Ma, 2021). The Blue Horse Café case in Turkey highlighted contrasts in relationships with healthcare workers versus relationships in a community-based therapeutic community, with descriptions of the former cold, indifferent or lacking in sincerity, whereas the humanistic aspects of the latter were detailed in terms of equal power relations and mutual respect. The space was described as supportive of power-sharing between health workers and people living with SMI (Soygür et al., 2017). However, not all healthcare workers might feel comfortable working outside the spheres of health facilities. In the reporting of the Banyan-supported housing model, healthcare workers experienced challenges adapting to their roles in community settings and social rather than biomedical orientation. There was also a cultural dimension, in that unmarried female healthcare workers felt pressure to justify them living unmarried in the community where they worked (Padmakar et al., 2020). In the Chinese case, a lack of role clarification for frontline workers attending to multiple vulnerable populations and working across sectors resulted in them experiencing increased pressure to deal with SMI. Also, the dominance of the Chinese government resulted in cooperation between government and social organisations being driven by the willingness of government organisations to work with social organisations (and not vice versa), thereby skewing the power differential towards government departments rooted in psychiatric professional expertise (Li and Ma, 2021). Nonetheless, intersectoral working was codified in formal arrangements, which is not the case in many other settings. In South Africa, there is a recognised need for input from social services, education and labour into recovery programmes (Gamieldien et al., 2022). Further, recommendations were made that the Department of Health, Department of Social Development and NGO sectors should improve communication between sectors, promote leadership from all levels and formalise intersectoral relationships through appropriate written agreements (Brooke-Sumner et al., 2016). The lack of formal agreements and intersectoral policy was also highlighted in Timor-Leste, which often translated into limited prioritising of mental healthcare (Hall et al., 2019). Finally, there are persistent structural barriers faced by organisations and individuals alike when pursuing SMI recovery in community settings. For instance, the Banyan model faced challenged from private property owners when attempting to secure housing for their beneficiaries, which included stigmatising attitudes towards people living with SMI and enacting a preference for residents who are more functional and mobile (Padmakar et al., 2020).

## Discussion

The aim of this scoping review was to identify and describe multi- and intersectoral approaches to enable SMI recovery in LMICs. The principal finding of this scoping review is that while such approaches have been widely supported in literature on developing appropriate and responsive support systems for SMI, very few studies operationalise and describe how multi-and intersectoral work is done in relation to recovery services in LMICs. This is in contrast to the comparatively vast body of work on multisectoral interorganizational collaboration and networks for community SMI care developed in HICs (Rosenheck et al., 1998; Fleury and Mercier, 2002; Morrissey et al., 2002; Wiktorowicz et al., 2010; Whiteford et al., 2014; Lorant et al., 2017; Jørgensen et al., 2020; Nicaise et al., 2021). From its origins from a WHO technical working group who realised that optimal sanitation requires a coordination between traditional public health and infectious disease actors and engineering and water management specialists (de Leeuw, 2022), intersectoral action has gained traction in global health discourse, though this has not been robustly translated to services supporting SMI recovery. Our review highlights several areas where multi-and intersectoral collaboration has been demonstrated in LMICs with respect to community support for people living with SMI, particularly in the areas of housing support, the development and sustainment of protective spaces where recovery can take place, and linkages between health facilities and community resources.

The Integrated Recovery Model posits that each individual has subjective recovery needs, centred around basic needs such as accommodation and employment, as well as less tangible needs such as coping skills and hope. Three core components interact with these needs: remediation of functioning (recovering mental and physical well-being), collaborative restoration of skills and competencies (building hope through collaborative restoration of agency, function and participation), and active community reconnection (re-establishing a place in the community with a range of skills and supports). Importantly, these processes unfold in linear and overlapping fashion. During the critical period following deterioration of well-being, remediation comes into play, where collaboration between various community actors and sectors and acute mental health services are critical. During the restoration period, psychosocial rehabilitation becomes central, which again necessitates the mobilisation of collaborations and resources in LMIC settings. Finally, moving towards an achievement of a degree of recovery, various community-based actors including NGOs, faith-based actors and community members becomes key (Frost et al., 2017). Our findings here suggest that, in most settings, elements of this model can be found, addressing the key movements from psychiatric relapse to recovery and community integration – whether through initial referral for specialist care, fulfilling basic needs or sustaining safe spaces and collaborations across sectors for people to recover.

Importantly, in many societies where tightly-knit families and high level of social cohesion are prevalent, especially in African and sub-Indian continental communities, the family has (and continues to be) a central locus of care beyond the boundaries of facility-based mental healthcare (Alem et al., 2008; Chadda, 2012). Family caregivers in LMIC are key to creating an environment that supports recovery but the burden of care is compounded by less-developed community systems of care, marked social stigma and certain cultural practices (Karambelas et al., 2022). People with SMI and their caregivers face barriers to securing formal income or employment, food, housing, transport and education (Addo et al., 2018). Holistic care for this vulnerable group is thus intricately linked with poverty alleviation, development and working towards social inclusion (Lund et al., 2011; Jenkins et al., 2011a, 2011b, 2011c; Plagerson, 2015) all of which have been hampered by the impact of COVID-19 (Kola et al., 2021).

Several studies indicate the leading coordinating role of non-governmental or charitable organisations (e.g., BasicNeeds) in bringing together stakeholders from other sectors for recovery-focused work. While this may be effective, NGOs are commonly reliant on donor funding and programmes may not be sustained in the long term and the corresponding influence in coordinating intersectoral action may be eroded. While a whole of government approach is indicated it is likely that one stakeholder or partner sector should take a leading coordinating role in sustaining intersectoral work and this need not be the health sector. In principle, this involves moving away from a purely biomedical model of treatment and recovery for SMI in which sectors other than the health sector recognise the role of the social environment in creating psychosocial disability associated with these conditions (World Health Organization, 2019). The benefits of such a shift also include sharing of the economic burden of SMI across sectors, a critical step away from health facility-focused spending (OECD, 2021). Although the literature on operationalised intersectoral and multisectoral work for recovery is limited, findings of this review suggest overarching domains for action that may be pursued (in context-specific ways) to drive intersectoral work in LMIC. These are: (1) building relationships between key actors including people with lived experience and families; (2) prioritising supportive spaces for recovery that help with fulfilling basic needs; (3) building leadership capacity among actors to solidify and formalise intersectoral work and (4) integrating resource allocation between actors to upderpin these approaches. Country-specific approaches to may also benefit from leapfrogging, that is, harnessing strategies previously used in intersectoral initiatives of disability movements and advocacy for treatment and care for HIV and TB in LMIC.

## Limitations

The main limitation of this scoping review was the exclusion of non-English language papers, and of grey literature. This approach was taken as a feasible first approach to scoping the literature on this topic. A future review should consider inclusion of grey literature, given the importance of the non-profit/non-government sector in provision of community-based services for recovery. This review also did not include searches specifically looking at social welfare payments and health insurance coverage for treatment costs (which are available in some LMIC and may be considered a form of intersectoral work). A further scoping review is in process that will cover this topic.

## Conclusion

Multi- and intersectoral collaboration lies at the heart of recovery – “medical solutions to social problems are expensive, ineffective and inefficient,” and integration between the biomedical and the social is “humane, cost-effective and truly recovery-oriented” (Drake and Whitley, 2014). In this review, we have described limited, though promising, examples of such action. This hopefully serves as a call for researchers, policymakers and service providers to both work more deliberately with other sectors and to strive to be more strategic in doing so, while keeping the recovery needs of the individual at the centre of actions.

## Considerations for the future

As the barriers outlined in the findings suggest, these are often piecemeal and relatively uncoordinated ventures, and require more deliberate, strategically coordinated actions. Multi- and intersectoral action for health and well-being involve strategies and action plans; long-term multisectoral and intersectoral initiatives; permanent structures; projects; legislative or parliamentary decisions and tools (World Health Organization, 2018). The following five recommendations have been suggested to facilitate intersectoral action for mental health in LMICs (Skeen et al., 2010) which align with the four domains for action described above:

1. Develop supportive legislation and policy alongside the other formalised structures for intersectoral action
2. Develop leadership in the health sector and beyond, especially in cross-cutting agencies
3. Employ targeted awareness-raising to engage all relevant sectors in order to specify roles, responsibilities and strategies
4. Develop a formal, structured approach to intersectoral action for mental health to address the lack of dedicated budgeting and unclear roles
5. Drive intersectoral work on a microlevel, in order to effectively address basic services such as water, electricity and sanitation

The principles for recovery-related service delivery should further be couched in these structures, including that services are person-centred, holistic and inclusive; enable agency and self-management; integrated across the care continuum; seamless and complementary across government departments, NGOs and other services; evidence-based; underlines equity in choosing service options; and are aligned with national, national and local strategy (Frost et al., 2017).

In terms of the findings reported here, generating universal recommendations or actions is challenging given the wide array of health and social systems across countries and regions. Nonetheless, there are thematic clusters that could provide direction to policymakers and other stakeholders in strengthening recovery efforts in an inter and multisectoral way. A common strategy that emerged relates to providing housing support, which ranged from communal, semi-institutionalised recovery settings to independent living in supportive housing arrangements. This requires the acquisition and appropriate management of physical spaces and require partnerships between the health sector, public civil infrastructure sector as well as private citizens and organisations, and formal arrangements through public–private partnerships should be set in place. Regarding community support systems, many examples here highlight linkages between people living with SMI and their caregivers, and various community-based resources. This requires a community-based body, organisation, clinic or government agency (depending on the health system configuration) that can set up relationships with and curate a list of a range of resources that people can be referred to. Especially in lower-resource settings, it is essential to tap into existing resources beyond the health sector that often remain underutilised in supporting people living with SMI. Lessons can be gleaned from many examples of multisectoral collaboration in addressing HIV, TB and non-communicable diseases. In terms of supportive spaces for recovery, many NGOs provide such environments with various types and degrees of support, much of which relates to assistance in navigating the bureaucracies involved in accessing grant schemes as well as supporting people to access the job market. This requires willing employers and a supportive employment work environment, which, given perpetuating stigma, would require educational investment as well as buy-in from appropriate employers. In terms of the bureaucracies of accessing grant schemes, more can be done by government agencies to remove administrative obstacles for people living with SMI, for example, making the medical diagnostic screening process more accessible. Finally, it is crucial to develop an appropriate health worker mix to deliver the complex range of activities within the ambit of recovery and inter- and multisectoral approaches. Task-sharing and empowering lay health workers have grown substantially as a viable option for constrained settings, and lay health workers can potentially offer crucial linkages with sectors and resources outside of the health sector. Nonetheless, these workers need appropriate training, regulation and a supportive career pathway in order to sustain their role in recovery-oriented services.

Finally, a particular challenge that emerged during the search and screen phases of this review was the imprecision evident in descriptions of multi-and intersectoral collaboration. Descriptions of the roles and remits of sectors were lacking, and are required to give context to the application of multi or intersectoral work. Intersectoral collaboration has been described as “an intricate web of interdependent organisations, individuals and behaviours, implicitly or explicitly driven by beliefs or assumptions to pursue a set of interconnected ideals, goals and objectives through the variously dispersed and joint control and allocation of resources” (de Leeuw, 2022). Given this complexity, an approach to render descriptions of multi- and intersectoral work more explicit could be for reviewers and journals involved in publishing recovery-based studies and interventions to request details on the ways that partnerships are formed and maintained (partnership working as a heading).

## Data Availability

Data are available on request from the corresponding author.

## Supplementary material

For supplementary material accompanying this paper visit https://doi.org/10.1017/gmh.2023.10. click here to view supplementary material

## References

[r1] Acebal JS, Barbosa GC, Domingos TDS, Bocchi SCM and Paiva ATU (2021) Living in psychosocial rehabilitation: Analysis between two residential therapeutic services. Saúde em Debate 44, 1120–1133.

[r2] Addo R, Agyemang SA, Tozan Y and Nonvignon J (2018) Economic burden of caregiving for persons with severe mental illness in Sub-Saharan Africa: A systematic review. PLoS One 13, e0199830. 30092073 10.1371/journal.pone.0199830 PMC6084810

[r3] Alem A, Jacobsson L and Hanlon C (2008) Community-based mental health care in Africa: Mental health workers’ views. World Psychiatry: Official Journal of the World Psychiatric Association (WPA) 7, 54–57. 18458779 10.1002/j.2051-5545.2008.tb00153.x PMC2327237

[r4] Alhadidi MM, Lim Abdullah K, Yoong TL, Al Hadid L and Danaee M (2020) A systematic review of randomized controlled trials of psychoeducation interventions for patients diagnosed with schizophrenia. International Journal of Social Psychiatry 66, 542–552. 32507073 10.1177/0020764020919475

[r5] Ali S, Santomauro D, Ferrari AJ and Charlson F (2022) Excess mortality in severe mental disorders: A systematic review and meta-regression. Journal of Psychiatric Research 149, 97–105. 35259666 10.1016/j.jpsychires.2022.02.036

[r6] Al-Sawafi A, Lovell K, Renwick L and Husain N (2020) Psychosocial family interventions for relatives of people living with psychotic disorders in the Arab world: Systematic review. BMC Psychiatry 20, 1–14. 32819316 10.1186/s12888-020-02816-5 PMC7441715

[r7] Anish K (2013) An evaluation of psychosocial rehabilitation facilities for homeless mentally ill in India. Artha Journal of Social Sciences 12, 1–19.

[r8] Arahanthabailu P, Purohith AN, Kanakode R, Praharaj SK, Bhandary RP and Venkata Narasimha Sharma PS (2022) Modified assertive community treatment program for patients with schizophrenia: Effectiveness and perspectives of service consumers from a South Indian setting. Asian Journal of Psychiatry 73, 103102. 35452965 10.1016/j.ajp.2022.103102

[r9] Arias D, Taylor L, Ofori-Atta A and Bradley EH (2016) Prayer camps and biomedical Care in Ghana: Is collaboration in mental Health care possible? PLoS One 11, e0162305. 27618551 10.1371/journal.pone.0162305 PMC5019394

[r10] Arksey H and O’malley L (2005) Scoping studies: Towards a methodological framework. International Journal of Social Research Methodology 8, 19–32.

[r11] Asher L, Birhane R, Weiss HA, Medhin G, Selamu M, Patel V, De Silva M, Hanlon C and Fekadu A (2022) Community-based rehabilitation intervention for people with schizophrenia in Ethiopia (RISE): Results of a 12-month cluster-randomised controlled trial. The Lancet Global Health 10, e530–e542. 35303462 10.1016/S2214-109X(22)00027-4 PMC8938762

[r12] Asher L, Fekadu A, Hanlon C, Mideksa G, Eaton J, Patel V and De Silva MJ (2015) Development of a community-based rehabilitation intervention for people with schizophrenia in Ethiopia. PLoS One 10, e0143572. 26618915 10.1371/journal.pone.0143572 PMC4664267

[r13] Asher L, Fekadu A, Teferra S, De Silva M, Pathare S and Hanlon C (2017) I cry every day and night, I have my son tied in chains: Physical restraint of people with schizophrenia in community settings in Ethiopia. Globalization and Health 13, 47. 28693614 10.1186/s12992-017-0273-1 PMC5504711

[r14] Bighelli I, Rodolico A, García-Mieres H, Pitschel-Walz G, Hansen W-P, Schneider-Thoma J, Siafis S, Wu H, Wang D and Salanti G (2021) Psychosocial and psychological interventions for relapse prevention in schizophrenia: A systematic review and network meta-analysis. The Lancet Psychiatry 8, 969–980. 34653393 10.1016/S2215-0366(21)00243-1

[r15] Brooke-Sumner C, Lund C and Petersen I (2014) Perceptions of psychosocial disability amongst psychiatric service users and caregivers in South Africa. African Journal of Disability 3, 146. 28730007 10.4102/ajod.v3i1.146 PMC5443050

[r16] Brooke-Sumner C, Lund C and Petersen I (2016) Bridging the gap: Investigating challenges and way forward for intersectoral provision of psychosocial rehabilitation in South Africa. International Journal of Mental Health Systems 10, 21. 26962328 10.1186/s13033-016-0042-1 PMC4784432

[r17] Brooke-Sumner C, Petersen I, Asher L, Mall S, Egbe CO and Lund C (2015) Systematic review of feasibility and acceptability of psychosocial interventions for schizophrenia in low and middle income countries. BMC Psychiatry 15, 1–12. 25886524 10.1186/s12888-015-0400-6 PMC4382830

[r18] Brooke-Sumner C, Selohilwe O, Mazibuko MS and Petersen I (2018) Process evaluation of a pilot intervention for psychosocial rehabilitation for service users with schizophrenia in North West Province, South Africa. Community Mental Health Journal 54, 1089–1096. 30094739 10.1007/s10597-018-0318-9

[r19] Chadda RK (2012) Six decades of community psychiatry in India. International Psychiatry: Bulletin of the Board of International Affairs of the Royal College of Psychiatrists 9, 45–47. 31508119 PMC6735056

[r20] Chatterjee S, Naik S, John S, Dabholkar H, Balaji M, Koschorke M, Varghese M, Thara R, Weiss HA and Williams P (2014) Effectiveness of a community-based intervention for people with schizophrenia and their caregivers in India (COPSI): A randomised controlled trial. The Lancet 383, 1385–1394. 10.1016/S0140-6736(13)62629-X PMC4255067 24612754

[r21] Chen Y, Yau E, Lam C, Deng H, Weng Y, Liu T and Mo X (2020) A 6-month randomized controlled pilot study on the effects of the clubhouse model of psychosocial rehabilitation with Chinese individuals with schizophrenia. Administration and Policy in Mental Health 47, 107–114. 31617138 10.1007/s10488-019-00976-5

[r22] Davies C, Radua J, Cipriani A, Stahl D, Provenzani U, Mcguire P and Fusar-Poli P (2018) Efficacy and acceptability of interventions for attenuated positive psychotic symptoms in individuals at clinical high risk of psychosis: A network meta-analysis. Frontiers in Psychiatry 9, 187. 29946270 10.3389/fpsyt.2018.00187 PMC6005890

[r23] De Leeuw E (2022) Intersectorality and health: A glossary. Journal of Epidemiology and Community Health 76, 206–208. 34706927 10.1136/jech-2021-217647 PMC8761990

[r24] De Menil V, Knapp M, Mcdaid D, Raja S, Kingori J, Waruguru M, Wood SK, Mannarath S and Lund C (2015) Cost-effectiveness of the mental Health and development model for schizophrenia-spectrum and bipolar disorders in rural Kenya. Psychological Medicine 45, 2747–2756. 25994212 10.1017/S0033291715000719

[r25] Diminic S, Carstensen G, Harris M, Reavley N, Pirkis J, Meurk C, Wong I, Bassilios B and Whiteford H (2015) Intersectoral policy for severe and persistent mental illness: Review of approaches in a sample of high-income countries. Global Mental Health 2, e18. 28596866 10.1017/gmh.2015.16 PMC5269620

[r26] Drake RE and Whitley R (2014) Recovery and severe mental illness: Description and analysis. Canadian Journal of Psychiatry 59, 236–242. 25007276 10.1177/070674371405900502 PMC4079142

[r27] Fleury M-J, Grenier G, Vallée C, Aubé D and Farand L (2017) Implementation of integrated service networks under the Quebec mental health reform: Facilitators and barriers associated with different territorial profiles. International Journal of Integrated Care 17, 3. 10.5334/ijic.2482 PMC5630082 29042845

[r28] Fleury M-J and Mercier C (2002) Integrated local networks as a model for organizing mental Health services. Administration and Policy in Mental Health and Mental Health Services Research 30, 55–73. 12546256 10.1023/a:1021227600823

[r29] Frederick DE and Vanderweele TJ (2019) Supported employment: Meta-analysis and review of randomized controlled trials of individual placement and support. PLoS One 14, e0212208. 30785954 10.1371/journal.pone.0212208 PMC6382127

[r30] Frost BG, Tirupati S, Johnston S, Turrell M, Lewin TJ, Sly KA and Conrad AM (2017) An integrated recovery-oriented model (IRM) for mental health services: Evolution and challenges. BMC Psychiatry 17, 22. 28095811 10.1186/s12888-016-1164-3 PMC5240195

[r31] Gamieldien F, Galvaan R, Myers B and Sorsdahl K (2022) Service providers perspectives on personal recovery from severe mental illness in Cape Town, South Africa: A qualitative study. Community Mental Health Journal 58, 955–966. 34671918 10.1007/s10597-021-00904-8 PMC9187550

[r32] Gamieldien F, Galvaan R, Myers B, Syed Z and Sorsdahl K (2021) Exploration of recovery of people living with severe mental illness (SMI) in low/middle-income countries (LMICs): A scoping review. BMJ Open 11, e045005. 10.1136/bmjopen-2020-045005 PMC7993175 33762242

[r33] Grazier KL, Mowbray CT and Holter MC (2005) Rationing psychosocial treatments in the United States. International Journal of Law and Psychiatry 28, 545–560. 16139889 10.1016/j.ijlp.2005.08.008

[r34] Hall T, Kakuma R, Palmer L, Minas H, Martins J and Armstrong G (2019) Intersectoral collaboration for people-centred mental health care in Timor–Leste: A mixed-methods study using qualitative and social network analysis. International Journal of Mental Health Systems 13, 72. 31788024 10.1186/s13033-019-0328-1 PMC6858633

[r35] İncedere A and Yildiz M (2019) Case Management for Individuals with severe mental illness: Outcomes of a 24-month practice. Turkish Journal of Psychiatry 30, 245–252. 32594485

[r36] Isaacs AN (2022) Care coordination as a collaborative element of recovery oriented services for persons with severe mental illness. Australasian Psychiatry 30, 110–112. 34464218 10.1177/10398562211037331

[r37] Janse Van Rensburg A, Petersen I, Wouters E, Engelbrecht M, Kigozi G, Fourie P, Van Rensburg D and Bracke P (2018) State and non-state mental health service collaboration in a South African district: A mixed methods study. Health Policy and Planning 33, 516–527. 29462292 10.1093/heapol/czy017

[r38] JBI (2015) Joanna Briggs Institute Reviewers’ Manual: Methodology for JBI Scoping Reviews. Adelaide: JBI.

[r39] Jenkins R, Baingana F, Ahmad R, Mcdaid D and Atun R (2011a) International and national policy challenges in mental health. Mental Health in Family Medicine 8, 101. 22654973 PMC3178192

[r40] Jenkins R, Baingana F, Ahmad R, Mcdaid D and Atun R (2011b) Mental health and the global agenda: Core conceptual issues. Mental Health in Family Medicine 8, 69–82. 22654969 PMC3178188

[r41] Jenkins R, Baingana F, Ahmad R, McDaid D and Atun R (2011c) Social, economic, human rights and political challenges to global mental health. Mental Health in Family Medicine 8, 87–96. 22654971 PMC3178190

[r42] Jørgensen K, Rasmussen T, Hansen M and Andreasson K (2021) Recovery-oriented intersectoral care between mental health hospitals and community mental health services: An integrative review. International Journal of Social Psychiatry 67, 788–800. 33100119 10.1177/0020764020966634

[r43] Jørgensen K, Rasmussen T, Hansen M, Andreasson K and Karlsson B (2020) Recovery-oriented intersectoral care in mental health: As perceived by healthcare professionals and users. International Journal of Environmental Research and Public Health 17, 8777. 33255970 10.3390/ijerph17238777 PMC7734578

[r44] Kallivayalil RA and Sudhakar S (2018) Effectiveness of a new low-cost psychosocial rehabilitative model to reduce burden of disease among persons with severe mental illness: An interventional follow-up study. Indian Journal of Psychiatry 60, 65–70. 29736065 10.4103/psychiatry.IndianJPsychiatry_85_17 PMC5914266

[r45] Karambelas GJ, Filia K, Byrne LK, Allott KA, Jayasinghe A and Cotton SM (2022) A systematic review comparing caregiver burden and psychological functioning in caregivers of individuals with schizophrenia spectrum disorders and bipolar disorders. BMC Psychiatry 22, 422. 35733174 10.1186/s12888-022-04069-w PMC9219207

[r46] Kohrt BA, Blasingame E, Compton MT, Dakana SF, Dossen B, Lang F, Strode P and Cooper J (2015) Adapting the crisis intervention Team (CIT) model of Police–Mental Health collaboration in a low-income, post-conflict country: Curriculum development in Liberia, West Africa. American Journal of Public Health 105, e73–e80. 25602903 10.2105/AJPH.2014.302394 PMC4330847

[r47] Kola L, Kohrt BA, Hanlon C, Naslund JA, Sikander S, Balaji M, Benjet C, Cheung EYL, Eaton J and Gonsalves P (2021) COVID-19 mental health impact and responses in low-income and middle-income countries: Reimagining global mental health. The Lancet Psychiatry 8, 535–550. 33639109 10.1016/S2215-0366(21)00025-0 PMC9764935

[r48] Lambert AM, Parretti HM, Pearce E, Price MJ, Riley M, Ryan R, Tyldesley-Marshall N, Avşar TS, Matthewman G, Lee A, Ahmed K, Odland ML, Correll CU, Solmi M and Marshall T (2022) Temporal trends in associations between severe mental illness and risk of cardiovascular disease: A systematic review and meta-analysis. PLoS Medicine 19, e1003960. 35439243 10.1371/journal.pmed.1003960 PMC9017899

[r49] Levac D, Colquhoun H and O’brien KK (2010) Scoping studies: Advancing the methodology. Implementation Science 5, 69. 20854677 10.1186/1748-5908-5-69 PMC2954944

[r50] Li Y and Ma H (2021) Interorganisational cooperation and its effects on community rehabilitation for people with severe mental disorders in Beijing, China: A case study. Health & Social Care in the Community 29, 154–163. 32627958 10.1111/hsc.13078

[r51] Lorant V, Nazroo J and Nicaise P (2017) Optimal network for patients with severe mental illness: A social network analysis. Administration and Policy in Mental Health 44, 877–887. 28341927 10.1007/s10488-017-0800-7 PMC5640746

[r52] Lund C, De Silva M, Plagerson S, Cooper S, Chisholm D, Das J, Knapp M and Patel V (2011) Poverty and mental disorders: Breaking the cycle in low-income and middle-income countries. The Lancet 378, 1502–1514. 10.1016/S0140-6736(11)60754-X 22008425

[r53] Lutgens D, Gariepy G and Malla A (2017) Psychological and psychosocial interventions for negative symptoms in psychosis: Systematic review and meta-analysis. The British Journal of Psychiatry 210, 324–332. 28302699 10.1192/bjp.bp.116.197103

[r54] Macdougall AG, Krupa T, Lysaght R, Mutiso V, Casey R, Le Ber MJ, Ruhara R, Price E, Kidd S and Ndetei DM (2022) The CREATE strategy of rehabilitation and recovery for mental illness in low resource settings: Development processes and evaluation from a proof of concept study in Kenya. International Journal of Mental Health 51, 32–60.

[r55] Mascayano F, Alvarado R, Andrews HF, Baumgartner JN, Burrone MS, Cintra J, Conover S, Dahl CM, Fader KM and Gorroochurn P (2022) A recovery-oriented intervention for people with psychosis: A pilot randomized controlled trial. Psychiatric Services 73, 1225–1231. 35678081 10.1176/appi.ps.202000843

[r56] Mascayano F, Alvarado R, Andrews HF, Jorquera MJ, Lovisi GM, Souza FM, Pratt C, Rojas G, Restrepo-Toro ME, Fader K, Gorroochurn P, Galea S, Dahl CM, Cintra J, Conover S, Burrone MS, Baumgartner JN, Rosenheck R, Schilling S, Sarução KR, Stastny P, Tapia E, Cavalcanti MT, Valencia E, Yang LH and Susser E (2019) Implementing the protocol of a pilot randomized controlled trial for the recovery-oriented intervention to people with psychoses in two Latin American cities. Cadernos de Saúde Pública 35, e00108018. 31066775 10.1590/0102-311X00108018 PMC6688497

[r57] Molchanova E (2014) Mental health rehabilitation in the Kyrgyz Republic: Official and indigenous models. Journal of Psychosocial Rehabilitation and Mental Health 1, 23–26.

[r58] Mondal S, Van Belle S and Maioni A (2021) Learning from intersectoral action beyond health: A meta-narrative review. Health Policy and Planning 36, 552–571. 33564855 10.1093/heapol/czaa163 PMC8128009

[r59] Morillo H, Lowry S and Henderson C (2022) Exploring the effectiveness of family-based interventions for psychosis in low-and middle-income countries: A systematic review. Social Psychiatry and Psychiatric Epidemiology 57, 1749–1769. 35699742 10.1007/s00127-022-02309-8 PMC9375736

[r60] Morrissey JP, Calloway MO, Thakur N, Cocozza J, Steadman HJ, Dennis D and ACCESS National Evaluation Team (2002) Integration of service systems for homeless persons with serious mental illness through the ACCESS program. Psychiatric Services 53, 949–957. 12161668 10.1176/appi.ps.53.8.949

[r61] Muhić M, Janković S, Sikira H, Slatina Murga S, Mcgrath M, Fung C, Priebe S and Džubur Kulenović A (2022) Multifamily groups for patients with schizophrenia: An exploratory randomised controlled trial in Bosnia and Herzegovina. Social Psychiatry and Psychiatric Epidemiology 57, 1357–1364. 35150309 10.1007/s00127-022-02227-9 PMC8853005

[r62] National Institute of Mental Health (2022) *Mental Illness* (Online). Available at https://www.nimh.nih.gov/health/statistics/mental-illness (accessed 14 November 2022).

[r63] Nicaise P, Grard A, Leys M, Van Audenhove C and Lorant V (2021) Key dimensions of collaboration quality in mental health care service networks. Journal of Interprofessional Care 35, 28–36. 31928444 10.1080/13561820.2019.1709425

[r64] Nuño R, Coleman K, Bengoa R and Sauto R (2012) Integrated care for chronic conditions: The contribution of the ICCC framework. Health Policy 105, 55–64. 22071454 10.1016/j.healthpol.2011.10.006

[r65] Nxumalo Ngubane S, Mcandrew S and Collier E (2019) The experiences and meanings of recovery for Swazi women living with “schizophrenia.” Journal of Psychiatric and Mental Health Nursing 26, 153–162. 31044474 10.1111/jpm.12520

[r66] OECD (2021) A New Benchmark for Mental Health Systems: Tackling the Social and Economic Costs of Mental Ill-Health, OECD Health Policy Studies. Paris, France: OECD Publishing.

[r67] Oni T, Mcgrath N, Belue R, Roderick P, Colagiuri S, May CR and Levitt NS (2014) Chronic diseases and multi-morbidity: A conceptual modification to the WHO ICCC model for countries in health transition. BMC Public Health 14, 575. 24912531 10.1186/1471-2458-14-575 PMC4071801

[r68] Onken SJ, Craig CM, Ridgway P, Ralph RO and Cook JA (2007) An analysis of the definitions and elements of recovery: A review of the literature. Psychiatric Rehabilitation Journal 31, 9. 17694711 10.2975/31.1.2007.9.22

[r69] Padmakar A, De Wit EE, Mary S, Regeer E, Bunders-Aelen J and Regeer B (2020) Supported housing as a recovery option for long-stay patients with severe mental illness in a psychiatric hospital in South India: Learning from an innovative de-hospitalization process. PLoS One 15, e0230074. 32271784 10.1371/journal.pone.0230074 PMC7144972

[r70] Patel V (2016) Universal health coverage for schizophrenia: A global mental health priority. Schizophrenia Bulletin 42, 885–890. 26245942 10.1093/schbul/sbv107 PMC4903041

[r71] Pfizer N and Kavitha M (2018) Psychosocial rehabilitation: A model by Rajah Rehabilitation Centre. Kerala Journal of Psychiatry 31, 38–45.

[r72] Plagerson S (2015) Integrating mental health and social development in theory and practice. Health Policy and Planning 30, 163–170. 24452138 10.1093/heapol/czt107

[r73] Raja S, Underhill C, Shrestha P, Sunder U, Mannarath S, Wood SK and Patel V (2012) Integrating mental health and development: A case study of the Basic needs model in Nepal. PLoS Medicine 9, e1001261. 22802741 10.1371/journal.pmed.1001261 PMC3393669

[r74] Rao K, John S, Kulandesu A, Karthick S, Senthilkumar S, Gunaselvi T, Raghavan V and Thara R (2022) Psychosocial rehabilitation of persons with severe mental disorders in rural South India: Learnings from step project. Journal of Psychosocial Rehabilitation and Mental Health 9, 335–343.

[r75] Rashed M (2015) From powerlessness to control: Psychosis, spirit possession and recovery in the Western desert of Egypt. Health, Culture and Society 8, 10–26.

[r76] Rodolico A, Bighelli I, Avanzato C, Concerto C, Cutrufelli P, Mineo L, Schneider-Thoma J, Siafis S, Signorelli MS and Wu H (2022) Family interventions for relapse prevention in schizophrenia: A systematic review and network meta-analysis. The Lancet Psychiatry 9, 211–221. 35093198 10.1016/S2215-0366(21)00437-5

[r77] Rosenheck R, Morrissey J, Lam J, Calloway M, Johnsen M, Goldman H, Randolph F, Blasinsky M, Fontana A and Calsyn R (1998) Service system integration, access to services, and housing outcomes in a program for homeless persons with severe mental illness. American Journal of Public Health 88, 1610–1615. 9807525 10.2105/ajph.88.11.1610 PMC1508580

[r78] Saha S, Chauhan A, Buch B, Makwana S, Vikar S, Kotwani P and Pandya A (2020) Psychosocial rehabilitation of people living with mental illness: Lessons learned from community-based psychiatric rehabilitation centres in Gujarat. Journal of Family Medicine and Primary Care 9, 892–897. 10.4103/jfmpc.jfmpc_991_19 PMC7114046 32318441

[r79] Sanni S, Wisdom JP, Ayo-Yusuf OA and Hongoro C (2019) Multi-sectoral approach to noncommunicable disease prevention policy in Sub-Saharan Africa: A conceptual framework for analysis. International Journal of Health Services 49, 371–392. 29745781 10.1177/0020731418774203

[r80] Silva P, Carvalho MCA, Cavalcanti MT, Echebarrena RC, Mello AS, Dahl CM, Lima DB and Souza FM (2017) Deinstitutionalization of long stay patients in a psychiatric hospital in Rio de Janeiro. Ciência & Saúde Coletiva 22, 2341–2352. 28724016 10.1590/1413-81232017227.19152015

[r81] Sin J and Spain D (2017) Psychological interventions for trauma in individuals who have psychosis: A systematic review and meta-analysis. Psychosis 9, 67–81.

[r82] Skeen S, Kleintjes S, Lund C, Petersen I, Bhana A, Flisher AJ and The Mental Health and Poverty Research Programme Consortium (2010) ‘Mental health is everybody’s business’: Roles for an intersectoral approach in South Africa. International Review of Psychiatry 22, 611–623. 21226649 10.3109/09540261.2010.535510

[r83] Solmi M, Croatto G, Piva G, Rosson S, Fusar-Poli P, Rubio JM, Carvalho AF, Vieta E, Arango C, DeTore NR, Eberlin ES, Mueser KT and Correll CU (2022) Efficacy and acceptability of psychosocial interventions in schizophrenia: Systematic overview and quality appraisal of the meta-analytic evidence. Molecular Psychiatry 28, 354–368. 35999275 10.1038/s41380-022-01727-z

[r84] Soygür H, Yüksel MM, Eraslan P and Attepe Özden S (2017) Lessons learned from experiencing Mavi at Café (blue horse Café) during six years: A qualitative analysis of factors contributing to recovery from the perspective of schizophrenia patients. Türk Psikiyatri Dergisi 28, 75–80. 29192939

[r85] Subandi M (2015) Bangkit: The processes of recovery from first episode psychosis in Java. Culture, Medicine, and Psychiatry 39, 597–613. 25600832 10.1007/s11013-015-9427-x

[r86] Tirfessa K, Lund C, Medhin G, Hailemichael Y, Fekadu A and Hanlon C (2019) Food insecurity among people with severe mental disorder in a rural Ethiopian setting: A comparative, population-based study. Epidemiology and Psychiatric Sciences 28, 397–407. 29143723 10.1017/S2045796017000701 PMC6998966

[r87] Tricco AC, Lillie E, Zarin W, O’brien KK, Colquhoun H, Levac D, Moher D, Peters MDJ, Horsley T, Weeks L, Hempel S, Akl EA, Chang C, Mcgowan J, Stewart L, Hartling L, Aldcroft A, Wilson MG, Garritty C, Lewin S, Godfrey CM, Macdonald MT, Langlois EV, Soares-Weiser K, Moriarty J, Clifford T, Tuncalp O and Straus SE (2018) PRISMA extension for scoping reviews (PRISMA-ScR): Checklist and explanation. Annals of Internal Medicine 169, 467–473. 30178033 10.7326/M18-0850

[r88] Vera San Juan N, Gronholm PC, Heslin M, Lawrence V, Bain M, Okuma A and Evans-Lacko S (2021) Recovery from severe mental Health problems: A systematic review of service user and informal caregiver perspectives. Frontiers in Psychiatry 12, 712026. 34539464 10.3389/fpsyt.2021.712026 PMC8440827

[r89] Vijayan T (2021) Psychiatric rehabilitation: A few recovery narratives. ASEAN Journal of Psychiatry 22(9), 1–9.

[r90] Warner R (2009) Recovery from schizophrenia and the recovery model. Current Opinion in Psychiatry 22, 374–380. 19417668 10.1097/YCO.0b013e32832c920b

[r91] Whiteford H, Mckeon G, Harris M, Diminic S, Siskind D and Scheurer R (2014) System-level intersectoral linkages between the mental health and non-clinical support sectors: A qualitative systematic review. Australian & New Zealand Journal of Psychiatry 48, 895–906. 25002710 10.1177/0004867414541683

[r92] Whitley R, Palmer V and Gunn J (2015) Recovery from severe mental illness. CMAJ 187, 951–952. 25918179 10.1503/cmaj.141558 PMC4577336

[r93] Wiktorowicz ME, Fleury M-J, Adair CE, Lesage A, Goldner E and Peters S (2010) Mental health network governance: Comparative analysis across Canadian regions. International Journal of Integrated Care 10, e60. 21289999 10.5334/ijic.525 PMC3031794

[r94] World Bank (2022) *Low & Middle Income* (Online). Available at https://data.worldbank.org/country/XO (accessed 14 November 2022).

[r95] World Health Organization (2018) *Multisectoral and Intersectoral Action for Improved Health and Well-Being for All: Mapping of the WHO European Region. Governance for a Sustainable Future: Improving Health and Well-Being for All.* World Health Organization. Regional Office for Europe.

[r96] World Health Organization (2019) WHO Thematic Brief on Mental Health: Multisectoral Action for Mental Health. Geneva: WHO.

[r97] World Health Organization (2021) Guidance on Community Mental Health Services: Promoting Person-Centred and Rights-Based Approaches. Geneva: World Health Organization.

[r98] World Health Organization (2022) World Mental Health Report: Transforming Mental Health for All. Geneva: WHO.

