# Supplementary material for: Intersectoral and multisectoral approaches to enable recovery for people with severe mental illness in low- and middle-income countries: A scoping review
Source: Glob Ment Health (Camb). 2023 Mar 15;10:e19. doi: 10.1017/gmh.2023.10 (PMC10579663; doi:10.1017/gmh.2023.10)
Supplement: Supplementary file 1 [file S2054425123000109sup001.docx]

**Addendum 1: Search terms**

| Search terms applied (LMIC search terms using EPOC LMIC filters 2020 (v.4) based on the World Bank list of economies 2019 (https://datahelpdesk.worldbank.org/knowledgebase/articles/906519-world-bank-country-and-lending-groups) |
| --- |
| ((((((Recovery[Title/Abstract] OR recover[Title/Abstract] OR psychosocial rehabilitation[Title/Abstract] OR mental health rehabilitation[Title/Abstract] OR psychiatric rehabilitation[Title/Abstract] OR mental health recovery[Title/Abstract] OR Recovery of function[Title/Abstract] OR Quality of life[Title/Abstract]) AND (Severe mental illness[Title/Abstract] OR bipolar[Title/Abstract] OR delusional disorder[Title/Abstract] OR delusional disorders[Title/Abstract] OR major depressive disorder[Title/Abstract] OR major depressive disorders[Title/Abstract] OR schizophrenia[Title/Abstract] OR manic[Title/Abstract] OR manic- depressive[Title/Abstract] OR paranoid disorder[Title/Abstract] OR paranoid disorders[Title/Abstract] OR psychoses[Title/Abstract] OR psychosis[Title/Abstract] OR psychotic disorder[Title/Abstract] OR psychotic disorders[Title/Abstract] OR schizoaffective disorder[Title/Abstract] OR schizoaffective disorders[Title/Abstract] OR Schizophreniform[Title/Abstract] OR serious mental disorder[Title/Abstract] OR serious mental disorders[Title/Abstract])) OR ("schizophrenia spectrum and other psychotic disorders"[MeSH Terms])) OR ("bipolar and related disorders"[MeSH Terms])) OR (depressive disorder, major[MeSH Terms])) AND ("low and middle income countries"[Title/Abstract])) AND (Deprived Countries[Title/Abstract] OR Deprived Population[Title/Abstract] OR Deprived Populations[Title/Abstract] OR Developing Countries[Title/Abstract] OR Developing Country[Title/Abstract] OR Developing Economies[Title/Abstract] OR Developing Economy[Title/Abstract] OR Developing Nation[Title/Abstract] OR Developing Nations[Title/Abstract] OR Developing Population[Title/Abstract] OR Developing Populations[Title/Abstract] OR Developing World[Title/Abstract] OR LAMI Countries[Title/Abstract] OR LAMI Country[Title/Abstract] OR Less Developed Countries[Title/Abstract] OR Less Developed Country[Title/Abstract] OR Less Developed Economies[Title/Abstract] OR Less Developed Nation[Title/Abstract] OR Less Developed Nations[Title/Abstract] OR Less Developed World[Title/Abstract] OR Lesser Developed Countries[Title/Abstract] OR Lesser Developed Nations[Title/Abstract] OR LMIC[Title/Abstract] OR LMICS[Title/Abstract] OR Low GDP[Title/Abstract] OR Low GNP[Title/Abstract] OR Low Gross Domestic[Title/Abstract] OR Low Gross National[Title/Abstract] OR Low Income Countries[Title/Abstract] OR Low Income Country[Title/Abstract] OR Low Income Economies[Title/Abstract] OR Low Income Economy[Title/Abstract] OR Low Income Nations[Title/Abstract] OR Low Income Population[Title/Abstract] OR Low Income Populations[Title/Abstract] OR Lower GDP[Title/Abstract] OR lower gross domestic[Title/Abstract] OR Lower Income Countries[Title/Abstract] OR Lower Income Country[Title/Abstract] OR Lower Income Nations[Title/Abstract] OR Lower Income Population[Title/Abstract] OR Lower Income Populations[Title/Abstract] OR Middle Income Countries[Title/Abstract] OR Middle Income Country[Title/Abstract] OR Middle Income Economies[Title/Abstract] OR Middle Income Nation[Title/Abstract] OR Middle Income Nations[Title/Abstract] OR Middle Income Population[Title/Abstract] OR Middle Income Populations[Title/Abstract] OR Poor Countries[Title/Abstract] OR Poor Country[Title/Abstract] OR Poor Economies[Title/Abstract] OR Poor Economy[Title/Abstract] OR Poor Nation[Title/Abstract] OR Poor Nations[Title/Abstract] OR Poor Population[Title/Abstract] OR Poor Populations[Title/Abstract] OR poor world[Title/Abstract] OR Poorer Countries[Title/Abstract] OR Poorer Economies[Title/Abstract] OR Poorer Economy[Title/Abstract] OR Poorer Nations[Title/Abstract] OR Poorer Population[Title/Abstract] OR Poorer Populations[Title/Abstract] OR Third World[Title/Abstract] OR Transitional Countries[Title/Abstract] OR Transitional Country[Title/Abstract] OR Transitional Economies[Title/Abstract] OR Transitional Economy[Title/Abstract] OR Under Developed Countries[Title/Abstract] OR Under Developed Country[Title/Abstract] OR under developed nations[Title/Abstract] OR Under Developed World[Title/Abstract] OR Under Served Population[Title/Abstract] OR Under Served Populations[Title/Abstract] OR Underdeveloped Countries[Title/Abstract] OR Underdeveloped Country[Title/Abstract] OR underdeveloped economies[Title/Abstract] OR underdeveloped nations[Title/Abstract] OR underdeveloped population[Title/Abstract] OR Underdeveloped World[Title/Abstract] OR Underserved Countries[Title/Abstract] OR Underserved Nations[Title/Abstract] OR Underserved Population[Title/Abstract] OR Underserved Populations[Title/Abstract])  PubMed 10/11/22  186 hits  After last 10 years applied: 167  After English applied: 167 |
| ((((((Recovery[Title/Abstract] OR recover[Title/Abstract] OR psychosocial rehabilitation[Title/Abstract] OR mental health rehabilitation[Title/Abstract] OR psychiatric rehabilitation[Title/Abstract] OR mental health recovery[Title/Abstract] OR Recovery of function[Title/Abstract] OR Quality of life[Title/Abstract]) AND (Severe mental illness[Title/Abstract] OR bipolar[Title/Abstract] OR delusional disorder[Title/Abstract] OR delusional disorders[Title/Abstract] OR major depressive disorder[Title/Abstract] OR major depressive disorders[Title/Abstract] OR schizophrenia[Title/Abstract] OR manic[Title/Abstract] OR manic- depressive[Title/Abstract] OR paranoid disorder[Title/Abstract] OR paranoid disorders[Title/Abstract] OR psychoses[Title/Abstract] OR psychosis[Title/Abstract] OR psychotic disorder[Title/Abstract] OR psychotic disorders[Title/Abstract] OR schizoaffective disorder[Title/Abstract] OR schizoaffective disorders[Title/Abstract] OR Schizophreniform[Title/Abstract] OR serious mental disorder[Title/Abstract] OR serious mental disorders[Title/Abstract])) OR ("schizophrenia spectrum and other psychotic disorders"[MeSH Terms])) OR ("bipolar and related disorders"[MeSH Terms])) OR (depressive disorder, major[MeSH Terms])) AND ("low and middle income countries"[Title/Abstract])) AND (Deprived Countries[Title/Abstract] OR Deprived Population[Title/Abstract] OR Deprived Populations[Title/Abstract] OR Developing Countries[Title/Abstract] OR Developing Country[Title/Abstract] OR Developing Economies[Title/Abstract] OR Developing Economy[Title/Abstract] OR Developing Nation[Title/Abstract] OR Developing Nations[Title/Abstract] OR Developing Population[Title/Abstract] OR Developing Populations[Title/Abstract] OR Developing World[Title/Abstract] OR LAMI Countries[Title/Abstract] OR LAMI Country[Title/Abstract] OR Less Developed Countries[Title/Abstract] OR Less Developed Country[Title/Abstract] OR Less Developed Economies[Title/Abstract] OR Less Developed Nation[Title/Abstract] OR Less Developed Nations[Title/Abstract] OR Less Developed World[Title/Abstract] OR Lesser Developed Countries[Title/Abstract] OR Lesser Developed Nations[Title/Abstract] OR LMIC[Title/Abstract] OR LMICS[Title/Abstract] OR Low GDP[Title/Abstract] OR Low GNP[Title/Abstract] OR Low Gross Domestic[Title/Abstract] OR Low Gross National[Title/Abstract] OR Low Income Countries[Title/Abstract] OR Low Income Country[Title/Abstract] OR Low Income Economies[Title/Abstract] OR Low Income Economy[Title/Abstract] OR Low Income Nations[Title/Abstract] OR Low Income Population[Title/Abstract] OR Low Income Populations[Title/Abstract] OR Lower GDP[Title/Abstract] OR lower gross domestic[Title/Abstract] OR Lower Income Countries[Title/Abstract] OR Lower Income Country[Title/Abstract] OR Lower Income Nations[Title/Abstract] OR Lower Income Population[Title/Abstract] OR Lower Income Populations[Title/Abstract] OR Middle Income Countries[Title/Abstract] OR Middle Income Country[Title/Abstract] OR Middle Income Economies[Title/Abstract] OR Middle Income Nation[Title/Abstract] OR Middle Income Nations[Title/Abstract] OR Middle Income Population[Title/Abstract] OR Middle Income Populations[Title/Abstract] OR Poor Countries[Title/Abstract] OR Poor Country[Title/Abstract] OR Poor Economies[Title/Abstract] OR Poor Economy[Title/Abstract] OR Poor Nation[Title/Abstract] OR Poor Nations[Title/Abstract] OR Poor Population[Title/Abstract] OR Poor Populations[Title/Abstract] OR poor world[Title/Abstract] OR Poorer Countries[Title/Abstract] OR Poorer Economies[Title/Abstract] OR Poorer Economy[Title/Abstract] OR Poorer Nations[Title/Abstract] OR Poorer Population[Title/Abstract] OR Poorer Populations[Title/Abstract] OR Third World[Title/Abstract] OR Transitional Countries[Title/Abstract] OR Transitional Country[Title/Abstract] OR Transitional Economies[Title/Abstract] OR Transitional Economy[Title/Abstract] OR Under Developed Countries[Title/Abstract] OR Under Developed Country[Title/Abstract] OR under developed nations[Title/Abstract] OR Under Developed World[Title/Abstract] OR Under Served Population[Title/Abstract] OR Under Served Populations[Title/Abstract] OR Underdeveloped Countries[Title/Abstract] OR Underdeveloped Country[Title/Abstract] OR underdeveloped economies[Title/Abstract] OR underdeveloped nations[Title/Abstract] OR underdeveloped population[Title/Abstract] OR Underdeveloped World[Title/Abstract] OR Underserved Countries[Title/Abstract] OR Underserved Nations[Title/Abstract] OR Underserved Population[Title/Abstract] OR Underserved Populations[Title/Abstract])  PubMed 10/11/22  186 hits  After last 10 years applied: 167  After English applied: 165 |
| AB ( recovery model or recovery approach or recovery framework ) AND AB ( mental health or mental illness or mental disorder or psychiatric illness or mental health crisis ) AND AB ( intersectoral collaboration or cooperative behavior or patient care team and primary health care and outcome and process assessment or healthcare multisectoral or multiprofessional or multidisciplinary ) AND AB ( emerging country OR emerging countries OR emerging nation OR emerging nations OR emerging population OR emerging populations developing country OR developing countries OR developing nation OR developing nations OR developing population OR developing populations OR developing world OR less developed country OR less developed countries OR less developed nation OR less developed nations OR less developed population OR less developed populations OR less developed world OR lesser developed country OR lesser developed countries OR lesser developed nation OR lesser developed nations OR lesser developed population OR lesser developed populations OR lesser developed world OR under developed country OR under developed countries OR under developed nation OR under developed nations OR under developed population OR under developed populations OR under developed world OR underdeveloped country OR underdeveloped countries OR underdeveloped nation OR underdeveloped nations OR underdeveloped population OR underdeveloped populations OR underdeveloped world OR middle income country OR middle income countries OR middle income nation OR middle income nations OR middle income population OR middle income populations OR low income country OR low income countries OR low income nation OR low income nations OR low income population OR low income populations OR lower income country OR lower income countries OR lower income nation OR lower income nations OR lower income population OR lower income populations OR underserved country OR underserved countries OR underserved nation OR underserved nations OR (underserved population OR underserved populations OR underserved world OR under served country OR under served countries OR under served nation OR under served nations OR under served population OR under served populations OR under served world OR deprived country OR deprived countries OR deprived nation OR deprived nations OR deprived population OR deprived populations OR deprived world OR poor country OR poor countries OR poor nation OR poor nations OR poor population OR poor populations OR poor world OR poorer country OR poorer countries OR poorer nation OR poorer nations OR poorer population OR poorer populations OR poorer world OR developing economy OR developing economies OR less developed economy OR less developed economies OR lesser developed economy OR lesser developed economies OR under developed economy OR under developed economies OR underdeveloped economy OR underdeveloped economies OR middle income economy OR middle income economies OR low income economy OR low income economies OR lower income economy OR lower income economies OR low gdp OR low gnp OR low gross domestic OR low gross national OR lower gdp OR lower gnp OR lower gross domestic OR lower gross national OR lmic OR lmics OR third world OR lami country OR lami countries OR transitional country OR transitional countries OR Africa OR Asia OR Caribbean OR West Indies OR South America OR Latin America OR Central America OR Atlantic Islands OR Pacific Islands OR Indian Ocean Islands OR Eastern Europe OR Afghanistan OR Albania OR Algeria OR Angola OR Antigua OR Barbuda OR Argentina OR Armenia OR Armenian OR Aruba OR Azerbaijan OR Bahrain OR Bangladesh OR Barbados OR Benin OR Byelarus OR Byelorussian OR Belarus OR Belorussian OR Belorussia OR Belize OR Bhutan OR Bolivia OR Bosnia OR Herzegovina OR Hercegovina OR Botswana OR Brasil OR Brazil OR Bulgaria OR Burkina Faso OR Burkina Fasso OR Upper Volta OR Burundi OR Urundi OR Cambodia OR Khmer Republic OR Kampuchea OR Cameroon OR Cameroons OR Cameron OR Camerons OR Cape Verde OR Central African Republic OR Chad OR Chile OR China OR Colombia OR Comoros OR Comoro Islands OR Comores OR Mayotte OR Congo OR Zaire OR Costa Rica OR Cote d'Ivoire OR Ivory Coast OR Croatia OR Cuba OR Cyprus OR Czechoslovakia OR Czech Republic OR Slovakia OR Slovak Republic OR Djibouti OR French Somaliland OR Dominica OR Dominican Republic OR East Timor OR East Timur OR Timor Leste OR Ecuador OR Egypt OR United Arab Republic OR El Salvador OR Eritrea OR Estonia OR Ethiopia OR Fiji OR Gabon OR Gabonese Republic OR Gambia OR Gaza OR Georgia Republic OR Georgian Republic OR Ghana OR Gold Coast OR Greece OR Grenada OR Guatemala OR Guinea OR Guam OR Guiana OR Guyana OR Haiti OR Honduras OR Hungary OR India OR Maldives OR Indonesia OR Iran OR Iraq OR Jamaica OR Jordan OR Kazakhstan OR Kazakh OR Kenya OR Kiribati OR Korea OR Kosovo OR Kyrgyzstan OR Kirghizia) OR (Kyrgyz Republic OR Kirghiz OR Kirgizstan OR Lao PDR OR Laos OR Latvia OR Lebanon OR Lesotho OR Basutoland OR Liberia OR Libya OR Lithuania OR Macedonia OR Madagascar OR Malagasy Republic OR Malaysia OR Malaya OR Malay OR Sabah OR Sarawak OR Malawi OR Nyasaland OR Mali OR Malta OR Marshall Islands OR Mauritania OR Mauritius OR Agalega Islands OR Melanesia OR Mexico OR Micronesia OR Middle East OR Moldova OR Moldovia OR Moldovian OR Mongolia OR Montenegro OR Morocco OR Ifni OR Mozambique OR Myanmar OR Myanma OR Burma OR Namibia OR Nepal OR Netherlands Antilles OR New Caledonia OR Nicaragua OR Niger OR Nigeria OR Northern Mariana Islands OR Oman OR Muscat OR Pakistan OR Palau OR Palestine OR Panama OR Paraguay OR Peru OR Philippines OR Philipines OR Phillipines OR Phillippines OR Poland OR Portugal OR Puerto Rico OR Romania OR Rumania OR Roumania OR Russia OR Russian OR Rwanda OR Ruanda OR Saint Kitts OR St Kitts OR Nevis OR Saint Lucia OR St Lucia OR Saint Vincent OR St Vincent OR Grenadines OR Samoa OR Samoan Islands OR Navigator Island OR Navigator Islands OR Sao Tome OR Saudi Arabia OR Senegal OR Serbia OR Montenegro OR Seychelles OR Sierra Leone OR Slovenia OR Sri Lanka OR Ceylon OR Solomon Islands OR Somalia OR Sudan OR Suriname OR Surinam OR Swaziland OR Syria OR Syrian OR Tajikistan OR Tadzhikistan OR Tadjikistan OR Tadzhik OR Tanzania OR Thailand OR Togo OR Togolese Republic OR Tonga OR Trinidad OR Tobago OR Tunisia OR Turkey OR Turkmenistan OR Turkmen OR Tuvalu OR Uganda OR Ukraine OR Uruguay OR USSR OR Soviet Union OR Soviet Socialist Republics OR Uzbekistan OR Uzbek OR Vanuatu OR New Hebrides OR Venezuela OR Vietnam OR Viet Nam OR West Bank OR Yemen OR Yugoslavia OR Zambia OR Zimbabwe OR Rhodesia OR Developing Countries OR Africa OR Asia OR Caribbean Region OR West Indies OR South America OR Latin America OR Central America OR Atlantic Islands OR Pacific Islands OR Indian Ocean Islands OR Europe, Eastern OR Afghanistan OR Albania OR Algeria OR American Samoa OR Angola OR Antigua and Barbuda OR Argentina OR Armenia OR Azerbaijan OR Bahrain OR Baltic States OR Bangladesh OR Barbados OR Benin OR Belarus OR Belize OR Bhutan OR Bolivia OR Bosnia-Herzegovina OR Botswana OR Brazil OR Bulgaria OR Burkina Faso OR Burundi OR Cambodia OR Cameroon OR Cape Verde OR Central African Republic OR Chad OR Chile OR China OR Colombia OR Comoros OR Congo OR Costa Rica OR Cote d'Ivoire OR Croatia OR Cuba OR Cyprus OR Czechoslovakia OR Czech Republic OR Slovakia OR Djibouti OR Congo OR Korea OR Dominica OR Dominican Republic OR East Timor OR Ecuador OR Egypt OR El Salvador OR Eritrea OR Estonia OR Ethiopia OR Equatorial Guinea OR Fiji OR French Guiana OR Gabon OR Gambia OR Georgia OR Ghana OR Greece OR Grenada OR Guatemala OR Guinea OR Guinea-Bissau OR Guam OR Guyana OR Haiti OR Honduras OR Hungary OR Samoa OR India OR Indonesia OR Iran OR Iraq OR Jamaica OR Jordan OR Kazakhstan OR Kenya OR Korea OR Kyrgyzstan OR Laos OR Latvia OR Lebanon OR Lesotho OR Liberia OR Libya OR Lithuania OR Macedonia OR Madagascar OR Malawi OR Malaysia OR Mali OR Malta OR Mauritania OR Mauritius OR Melanesia OR Mexico OR Micronesia OR Middle East OR Moldova OR Mongolia OR Montenegro OR Morocco OR Mozambique OR Myanmar OR Namibia OR Nepal OR Netherlands Antilles OR New Caledonia OR Nicaragua OR Niger OR Nigeria OR Oman OR Pakistan OR Palau OR Panama OR Papua New Guinea OR Paraguay OR Peru OR Philippines OR Poland OR Portugal OR Puerto Rico OR Romania OR Russia OR Russia OR Rwanda OR Saint Kitts and Nevis OR Saint Lucia OR Grenadines OR Samoa OR Saudi Arabia OR Senegal OR Serbia OR Montenegro OR Seychelles OR Sierra Leone OR Slovenia OR Sri Lanka OR Somalia OR South Africa OR Sudan OR Suriname OR Swaziland OR Syria OR Tajikistan OR Tanzania OR Thailand OR Togo OR Tonga OR Trinidad and Tobago OR Tunisia OR Turkey OR Turkmenistan OR Uganda OR Ukraine OR Uruguay OR USSR OR Uzbekistan OR Vanuatu OR Venezuela OR Vietnam OR Yemen OR Yugoslavia OR Zambia OR Zimbabwe OR Southern African Development Community OR East African Community OR West African Health Organisation )  Interface - EBSCOhost Research Databases  Search Screen - Advanced Search  Database - Academic Search Complete; APA PsycInfo; Health Source - Consumer Edition; Health Source: Nursing/Academic Edition; MasterFILE Premier; MEDLINE; OpenDissertations  Results: 13  Results after 10 years applied: 13  Results after English applied: 13 |
| (((("Recovery"[Title/Abstract] OR "recover"[Title/Abstract] OR "psychosocial rehabilitation"[Title/Abstract] OR "mental health rehabilitation"[Title/Abstract] OR "psychiatric rehabilitation"[Title/Abstract] OR "mental health recovery"[Title/Abstract] OR "recovery of function"[Title/Abstract] OR "quality of life"[Title/Abstract]) AND ("severe mental illness"[Title/Abstract] OR "bipolar"[Title/Abstract] OR "delusional disorder"[Title/Abstract] OR "delusional disorders"[Title/Abstract] OR "major depressive disorder"[Title/Abstract] OR "major depressive disorders"[Title/Abstract] OR "schizophrenia"[Title/Abstract] OR "manic"[Title/Abstract] OR "manic depressive"[Title/Abstract] OR "paranoid disorder"[Title/Abstract] OR "paranoid disorders"[Title/Abstract] OR "psychoses"[Title/Abstract] OR "psychosis"[Title/Abstract] OR "psychotic disorder"[Title/Abstract] OR "psychotic disorders"[Title/Abstract] OR "schizoaffective disorder"[Title/Abstract] OR "schizoaffective disorders"[Title/Abstract] OR "Schizophreniform"[Title/Abstract] OR "serious mental disorder"[Title/Abstract] OR "serious mental disorders"[Title/Abstract])) OR "schizophrenia spectrum and other psychotic disorders"[MeSH Terms] OR "bipolar and related disorders"[MeSH Terms] OR "depressive disorder, major"[MeSH Terms]) AND "low and middle income countries"[Title/Abstract] AND ("deprived countries"[Title/Abstract] OR "deprived population"[Title/Abstract] OR "deprived populations"[Title/Abstract] OR "developing countries"[Title/Abstract] OR "developing country"[Title/Abstract] OR "developing economies"[Title/Abstract] OR "developing economy"[Title/Abstract] OR "developing nation"[Title/Abstract] OR "developing nations"[Title/Abstract] OR "developing population"[Title/Abstract] OR "developing populations"[Title/Abstract] OR "developing world"[Title/Abstract] OR "lami countries"[Title/Abstract] OR "lami country"[Title/Abstract] OR "less developed countries"[Title/Abstract] OR "less developed country"[Title/Abstract] OR "less developed economies"[Title/Abstract] OR "less developed nation"[Title/Abstract] OR "less developed nations"[Title/Abstract] OR "less developed world"[Title/Abstract] OR "lesser developed countries"[Title/Abstract] OR "lesser developed nations"[Title/Abstract] OR "LMIC"[Title/Abstract] OR "LMICS"[Title/Abstract] OR "low gdp"[Title/Abstract] OR "low gnp"[Title/Abstract] OR "low gross domestic"[Title/Abstract] OR "low gross national"[Title/Abstract] OR "low income countries"[Title/Abstract] OR "low income country"[Title/Abstract] OR "low income economies"[Title/Abstract] OR "low income economy"[Title/Abstract] OR "low income nations"[Title/Abstract] OR "low income population"[Title/Abstract] OR "low income populations"[Title/Abstract] OR "lower gdp"[Title/Abstract] OR "lower gross domestic"[Title/Abstract] OR "lower income countries"[Title/Abstract] OR "lower income country"[Title/Abstract] OR "lower income nations"[Title/Abstract] OR "lower income population"[Title/Abstract] OR "lower income populations"[Title/Abstract] OR "middle income countries"[Title/Abstract] OR "middle income country"[Title/Abstract] OR "middle income economies"[Title/Abstract] OR "middle income nation"[Title/Abstract] OR "middle income nations"[Title/Abstract] OR "middle income population"[Title/Abstract] OR "middle income populations"[Title/Abstract] OR "poor countries"[Title/Abstract] OR "poor country"[Title/Abstract] OR "poor economies"[Title/Abstract] OR "poor economy"[Title/Abstract] OR "poor nation"[Title/Abstract] OR "poor nations"[Title/Abstract] OR "poor population"[Title/Abstract] OR "poor populations"[Title/Abstract] OR "poor world"[Title/Abstract] OR "poorer countries"[Title/Abstract] OR "poorer economies"[Title/Abstract] OR "poorer economy"[Title/Abstract] OR "poorer nations"[Title/Abstract] OR "poorer population"[Title/Abstract] OR "poorer populations"[Title/Abstract] OR "third world"[Title/Abstract] OR "transitional countries"[Title/Abstract] OR "transitional country"[Title/Abstract] OR "transitional economies"[Title/Abstract] OR "transitional economy"[Title/Abstract] OR "under developed countries"[Title/Abstract] OR "under developed country"[Title/Abstract] OR "under developed nations"[Title/Abstract] OR "under developed world"[Title/Abstract] OR "under served population"[Title/Abstract] OR "under served populations"[Title/Abstract] OR "underdeveloped countries"[Title/Abstract] OR "underdeveloped country"[Title/Abstract] OR "underdeveloped economies"[Title/Abstract] OR "underdeveloped nations"[Title/Abstract] OR "underdeveloped population"[Title/Abstract] OR "underdeveloped world"[Title/Abstract] OR "underserved countries"[Title/Abstract] OR "underserved nations"[Title/Abstract] OR "underserved population"[Title/Abstract] OR "underserved populations"[Title/Abstract]) AND ("intersectoral"[Title/Abstract] OR "multisectoral"[Title/Abstract])) AND ((y_10[Filter]) AND (english[Filter])) |
| 10/11/22  PubMed:  4 results  After 10 years: 4  After English: 4 |
| Interface - EBSCOhost Research Databases  Search Screen - Advanced Search  Database - Academic Search Complete; APA PsycInfo; Health Source - Consumer Edition; Health Source: Nursing/Academic Edition; MasterFILE Premier; MEDLINE; OpenDissertations  SU ( schizophrenia or psychosis or psychoses or psychotic disorder or schizophrenic disorder ) AND SU community AND SU ( intersectoral collaboration or cooperative behavior or patient care team and primary health care and outcome and process assessment, healthcare )  Limiters - Published Date: 20120101-20221231  Expanders - Apply equivalent subjects  Results: 51  Results after 10 years applied: 51  Results after English applied: 51 |
